# Proteomic Analysis of Excretory-Secretory Products of *Mesocestoides corti* Metacestodes Reveals Potential Suppressors of Dendritic Cell Functions

**DOI:** 10.1371/journal.pntd.0005061

**Published:** 2016-10-13

**Authors:** Emilia Vendelova, Jeferson Camargo de Lima, Karina Rodrigues Lorenzatto, Karina Mariante Monteiro, Thomas Mueller, Jyotishman Veepaschit, Clemens Grimm, Klaus Brehm, Gabriela Hrčková, Manfred B. Lutz, Henrique B. Ferreira, Justin Komguep Nono

**Affiliations:** 1 Institute of Parasitology of the Slovak Academy of Sciences, Košice, Slovak Republic; 2 Laboratório de Genômica Estrutural e Funcional, Centro de Biotecnologia, Universidade Federal do Rio Grande do Sul (UFRGS), Porto Alegre, Rio Grande do Sul, Brazil; 3 Departamento de Biologia Molecular e Biotecnologia, Instituto de Biociências, (UFRGS), Porto Alegre, Rio Grande do Sul, Brazil; 4 Lehrstuhl für Molekulare Pflanzenphysiologie und Biophysik, Julius-von-Sachs Institut der Universität Würzburg, Würzburg, Germany; 5 Lehrstuhl für Biochemie, Biozentrum der Universität Würzburg, Würzburg, Germany; 6 University of Würzburg, Institute for Hygiene and Microbiology, Würzburg, Germany; 7 University of Würzburg, Institute of Virology and Immunobiology, Würzburg, Germany; 8 Institute of Infectious Diseases and Molecular Medicine (IDM), Division of Immunology, University of Cape Town, Cape Town, South Africa; 9 Institute of Medical Research and Medicinal Plant Studies (IMPM), Ministry of Scientific Research and Innovation, Yaoundé, Cameroon; Instituto de Investigaciones Biomédicas, UNAM, MEXICO

## Abstract

Accumulating evidences have assigned a central role to parasite-derived proteins in immunomodulation. Here, we report on the proteomic identification and characterization of immunomodulatory excretory-secretory (ES) products from the metacestode larva (tetrathyridium) of the tapeworm *Mesocestoides corti (syn*. *M*. *vogae)*. We demonstrate that ES products but not larval homogenates inhibit the stimuli-driven release of the pro-inflammatory, Th1-inducing cytokine IL-12p70 by murine bone marrow-derived dendritic cells (BMDCs). Within the ES fraction, we biochemically narrowed down the immunosuppressive activity to glycoproteins since active components were lipid-free, but sensitive to heat- and carbohydrate-treatment. Finally, using bioassay-guided chromatographic analyses assisted by comparative proteomics of active and inactive fractions of the ES products, we defined a comprehensive list of candidate proteins released by *M*. *corti* tetrathyridia as potential suppressors of DC functions. Our study provides a comprehensive library of somatic and ES products and highlight some candidate parasite factors that might drive the subversion of DC functions to facilitate the persistence of *M*. *corti* tetrathyridia in their hosts.

## Author Summary

The metacestode larval stages of life-threatening tapeworms grow within the organs of its mammalian hosts, thus causing severe and long-lasting morbidity. Immunosuppression, which mainly depends on factors that are released or leaking from the parasite, plays an important role in both survival and proliferation of the larvae. These parasite-derived molecules are potential targets for developing new anti-parasitic drugs and/or improving the effectiveness of current therapies. Moreover, an optimized use of such factors could help to minimize pathologies resulting from uncontrolled immune responses, like allergies and autoimmune diseases. The authors herein demonstrate that larvae from a parasitic cestode release factors that sufficiently support the suppression of dendritic cells, a set of innate immune cells that recognizes and initiates host immune responses against invading pathogens. Employing modern analytic proteomic tools combined with immunological bioassays, several cestode-derived candidate immunomodulators were identified. This is the first bioassay-guided comprehensive library of candidate immunomodulators from a tissue-dwelling cestode larva. This work validates the unmet value of the *Mesocestoides corti* system in characterizing the mechanisms of host immunomodulation by metacestodes and reveals the largest database of candidate metacestode-derived immunomodulators until date.

## Introduction

Cestodes in general and the metacestode larval stages in particular are of major etiological importance for both human and domestic animal diseases. Currently available therapies against the deadliest metacestode-mediated diseases are still limited. Major life-threatening human cestodes such as *T*. *solium*, *E*. *granulosus* and *E*. *multilocularis* cause serious diseases due to the unique ability of their metacestode larvae to persist within the host tissues for decades, gradually impairing the function of the colonized organ [1,2]. Metacestodes dwell in the host tissues where they confront the immune system and modulate the immune response to enable their survival and the establishment of a chronic infection [3]. Therefore, severe pathology in mammalian hosts occurs late after long asymptomatic or subclinical infection periods, with little inflammatory responses or overt tissue destruction [1,2].

Cestodes, as most of the helminths, induce modified T-helper (Th) 2 immune responses that are accompanied by various immunoregulatory mechanisms to control excessive Th1 immunity that would prevent parasite colonization [2– 6]. The major factor instructing Th1 cell generation is the cytokine IL-12p70 released by dendritic cells (DCs) [4]. Thus, for tissue-dwelling metacestodes, interference with IL-12 production by DC is critical for limiting pro-inflammatory Th1 immunity and allowing parasite persistence [2,5–10]. However, how metacestodes modulate DC functions is largely unclear [2].

Excretory-secretory (ES) products of metacestodes are instrumental in the mitigation of IL-12 production by host DCs [2,11]. As such, metacestode ES products are attractive targets to understand the mechanisms governing host-parasite interactions since these products directly interact with host immune cells where they drive immunoregulation [2,7,9,11–14]. Proteomic analytical tools including mass spectrometry have helped the identification of ES products from *in vitro* cultures of parasitic helminths and led to the identification of candidate host protective antigens and immunomodulators alike [15–21]. As for the disease-mediating larvae of parasitic cestodes i.e. metacestodes, a major drawback was the dependency of all established culture systems on supplements from mammalian hosts [22] making it difficult to perform downstream proteomic analyses on metacestode culture supernatants. We recently developed an *in vitro* cultivation system for metacestodes (tetrathyridia) of the parasitic cestode *Mesocestoides corti* [22]. Our cultivation system enabled the collection of *M*. *corti* tetrathyridia ES products in medium devoid of host cells and other supplements such as serum [22]. Although axenic ES products of *M*. *corti* tetrathyridia isolated from our cultivation system sufficiently recapitulated *M*. *corti* tetrathyridia ability to suppress LPS-driven IL-12 production by DC *in vitro* [22], the molecular bases of DC suppression by ES products of *M*. *corti* tetrathyridia in particular and metacestodes in general still remains unknown.

In this study, we took advantage of our *M*. *corti* tetrathyridia cultivation system to characterize the DC suppressing effect of *M*. *corti* tetrathyridia ES products (McES). *In vitro* exposure of BMDCs to McES impaired their subsequent responsiveness to other pathogen products including ligands for TLRs and C-type lectins. The production of IL-12p70 from LPS-activated BMDCs was significantly reduced upon exposure to McES whereas exposure to *M*. *corti* tetrathyridia homogenates (McH) could not impair BMDC activation. Biochemical analyses of *M*. *corti* tetrathyridia ES products (McES) narrowed down the immunosuppressive activity to glycoproteins. Further analyzing McES by bioassay-guided fractionation assisted with liquid chromatography-mass spectrometry, we identified a set of candidate proteins that might mediate *M*. *corti* tetrathyridia suppression of DCs. Once functionally tested, this comprehensive library of metacestode-derived candidate immunomodulatory proteins should improve our understanding of how tissue-dwelling metacestodes subvert the host DC response.

## Results

### *M*. *corti* tetrathyridia attract DCs in experimentally infected mice

**Fig 1:**
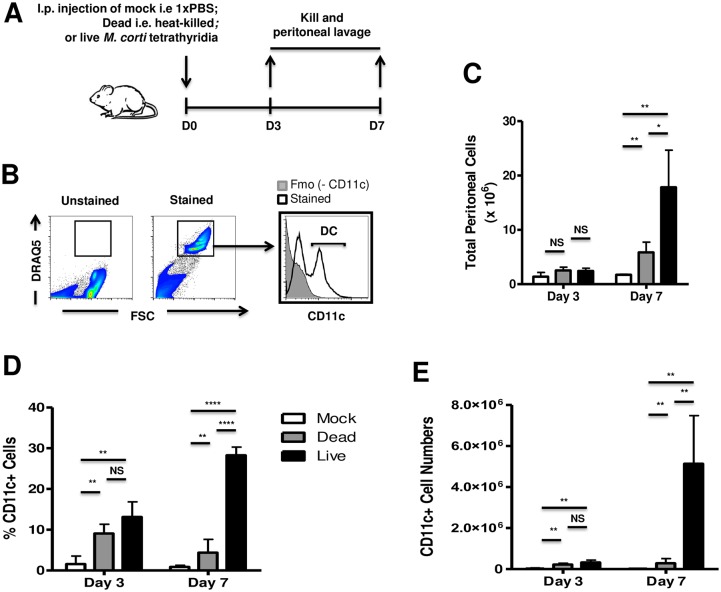
*M*. *corti* metacestode larvae (tetrathyridia) elicit a significant recruitment of host CD11c+ cells *in vivo*. (A) Schematic showing experimental set-up. (B) Gating strategy for CD11c+ cells in the peritoneal exudate cells. (C) Total peritoneal cells from mock–injected mice and mice injected i.p with heat-killed or live *M*.*corti* larvae were determined at day 3 and day 7 following injection. (D) Percentages of peritoneal CD11c+ cells at day 3 and day 7 post-injection. (E) Total peritoneal CD11c+ cell numbers at day 3 and day 7 post-injection. Data are means ± SD of values from 4–5 mice per group assayed individually. p>0.05, NS; p<0.05, *; p<0.01, **; p<0.001, ***; p≤0.0001, ****.

Having previously shown that *M*. *corti* impairs DC responsiveness to stimuli [22], we now sought to ascertain the *in vivo* relevance of DCs in the host response to *M*. *corti* tetrathyridia. To address this, we injected either live, heat-killed *M*. *corti* tetrathyridia or the PBS carrier solution (mock, negative control) into the peritoneum of BALB/c mice (Fig 1A) and analyzed the frequency of host CD11c^+^ cells within the total peritoneal exudate cells (Fig 1B). We found that viable tetrathyridia significantly recruited cells within the peritoneum at day 7 p.i. when compared to dead tetrathyridia or to the mock control (3-fold more than the dead larvae and 15-fold more than the mock injections) (Fig 1C). Further analyses showed that live *M*. *corti* tetrathyridia recruited growing proportion of CD11c+ (12% of the peritoneal exudate cells at day 3 p.i. and 27% at day 7 p.i.) (Fig 1D and 1E). In contrast, cells recruited less efficiently by heat-killed *M*. *corti* tetrathyridia harbored fewer CD11c^+^ host cells over time (10% of the peritoneal exudate cells at day 3 p.i. and 4% at day 7 p.i.) (Fig 1D and 1E). These results indicate that only live *M*. *corti* tetrathyridia massively attracted host CD11c+ cells up to day 7 post-infection whereas dead (ametabolic) larvae recruited CD11c+ cells just for the 3 days that followed injection. Given the ability of live helminths to excrete-secrete molecules which directly interact with host immune cells [11,23] and considering the reported predominance of dendritic cells within murine peritoneal CD11c+ cells, our data suggest a central role for ES products of *M*. *corti* (McES) in the modulation of host DC responses *in vivo*.

### McES limit TLR-driven BMDC activation and attenuate the response of activated BMDCs *in vitro*

**Fig 2:**
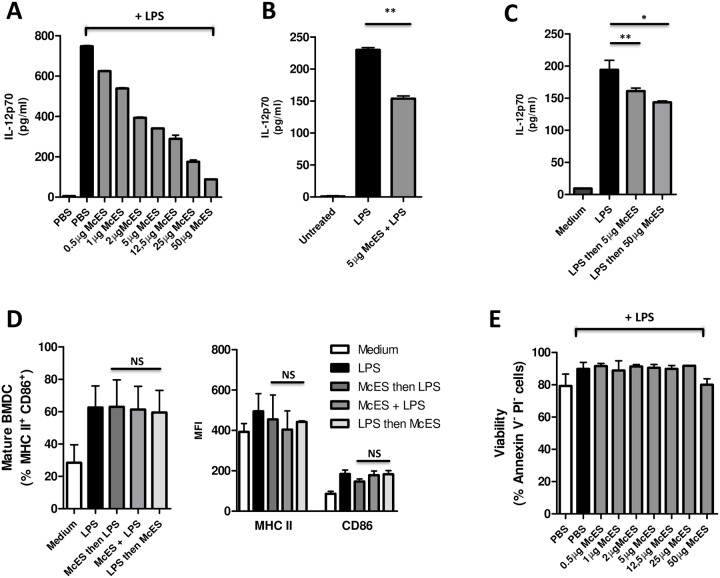
McES suppress BMDC activation. BMDCs were generated *in vitro* from C57BL/6 mice bone marrow cells and stimulated *in vitro* with different stimuli. 24 h later, BMDCs were restimulated with LPS and IL-12p70 was measured in the supernatant. (A) LPS-driven IL-12p70 release by BMDCs pre-treated with *M*. *corti* tetrathyridia ES products. (B) IL-12p70 release by BMDCs after simultaneous stimulation with LPS and *M*. *corti* ES products. (C) IL-12p70 release following restimulation of LPS-pre-treated BMDCs with *M*.*corti* ES products. (D) DC surface activation markers, MHCII and CD86 following different stimulation schemes with 5ug/ml of McES. Mean frequencies of Mature (MHCII+CD86+) DCs (left) and DC-specific MFI of MHCII and CD86 (right) are summarized. (E) Viability of BMDCs stimulated with different doses of *M*. *corti* ES products and LPS. The data are representative of 2–3 independent experiments. p>0.05, NS; p<0.05, *; p<0.01, **; p<0.001, ***.

To characterize the mechanisms of DC modulation by McES, BMDCs were exposed to a wide range of McES concentrations (0.5–50μg/ml) for 24h before subsequent stimulation with LPS for another 24h. After incubation, the culture supernatants were harvested and IL-12p70 production was measured by ELISA. As shown in Fig 2A, we found that all tested McES concentrations significantly inhibited LPS-driven release of IL-12p70 by BMDCs in a dose-dependent manner.

To rule out the possibility that McES bind to TLR-4 receptors to prevent the subsequent binding of LPS by steric hindrance, we assessed how the timing of exposure of host DCs to McES might influence the inhibitory effect on IL-12p70 production. In a first series of experiments, we simultaneously stimulated BMDCs with McES and LPS for 24h *in vitro* and measure the levels of LPS-driven IL-12p70 production. We noted that LPS-driven IL-12p70 production by BMDCs was significantly reduced in culture concomitantly supplemented with McES plus LPS (Fig 2B). In a second series of experiments, BMDCs were first activated with LPS for 24h and different doses of McES were then added to the activated BMDCs cultures for another 24h. We noted a significant and dose-dependent ability of McES to neutralize IL-12p70 release by LPS-activated BMDCs (Fig 2C). Fig 2D shows that BMDC upregulation of surface activation markers (MHCII and CD86) in response to LPS treatment was not affected by McES. To rule out any cytotoxic effect as a result of dual stimulation [24], BMDCs dually stimulated with different doses of McES and LPS were analyzed by Annexin-V/Propidium Iodide dual staining to identify viable cells with uncompromised cell membranes (Annexin-V^-^/Propidium Iodide^-^). BMDCs dually exposed to McES and LPS did not show any reduction in cell viability (Fig 2E) ruling out cell death as a possible cause of the reduced IL-12p70 production by BMDCs. These results demonstrated that McES limited LPS-driven BMDC activation independently of the time of exposure and also diminished the effector response (IL-12p70 production) of LPS-activated BMDCs *in vitro*. Conclusively, these findings indicate that McES suppressive effect is not merely a result of steric hindrance of the TLR-4 receptor on DCs.

### McES broadly impair BMDC responsiveness to different stimuli

**Fig 3:**
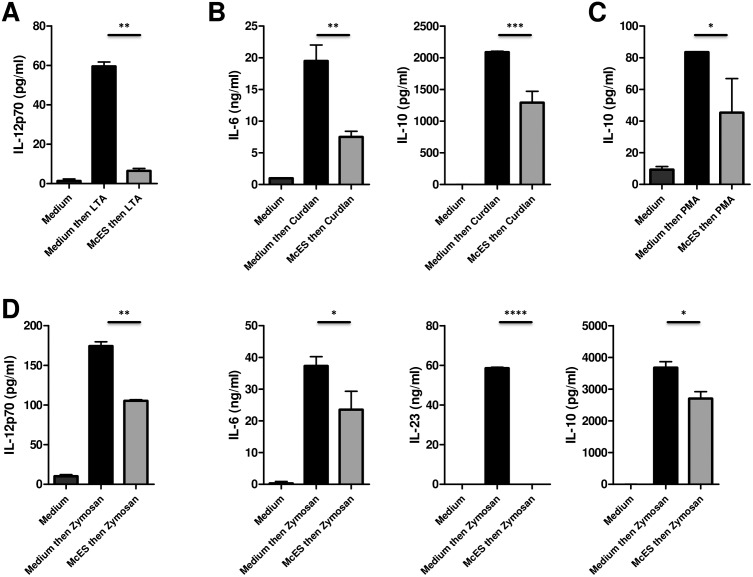
McES have a broad suppressive activity on BMDCs. BMDCs were generated *in vitro* from C57BL/6 mice bone marrow cells and stimulated *in vitro* with 5 μg/ml of *M*. *corti* ES products for 24 h before restimulation with various commercially available activation stimuli. Cytokine release following BMDC restimulation with (A) Lipoteichoic Acid (LTA), (B) Curdlan, (C) Phorbol myristate acetate (PMA) and (D) Zymosan. The data are representative of 2–4 independent experiments. p>0.05, NS; p<0.05, *; p<0.01, **; p<0.001, ***; p≤0.0001, ****.

We extended our studies by investigating the effect of McES on BMDC ability to respond to other activation stimuli i.e. *Staphylococcus aureus* lipoteichoic acid (LTA), a TLR-2 agonist; *Alcaligenes faecalis* Beta-1,3-glucan (Curdlan), a Dectin-1 agonist; *Saccharomyces cerevisiae* cell wall extract (Zymosan A), a dual TLR-2 and dectin-1 agonist and phorbol 12-myristate 13-acetate (PMA), an activator of protein kinase C and NF-κB by measuring the release of IL-12p70, IL-10, IL-6 and IL-23 into the culture supernatants. Since activation of immature DCs via different routes leads to distinct cytokine profiles [25,26], we used the cytokine(s) most abundantly produced by each stimulus to better capture the possible suppressive effect of McES. As shown in Fig 3, pre-exposure of BMDCs to McES significantly reduced LTA-driven IL-12p70 release (Fig 3A), curdlan-driven IL-6 and IL-10 secretions (Fig 3B), PMA-driven IL-10 secretion (Fig 3C) and zymosan-driven IL-12p70, IL-6, IL-23 and IL-10 secretions (Fig 3D). These results indicated that McES broadly impair BMDCs activation by various stimulatory ligands.

### Glycoproteins are the mediators of the immunosuppressive effects by McES

**Fig 4:**
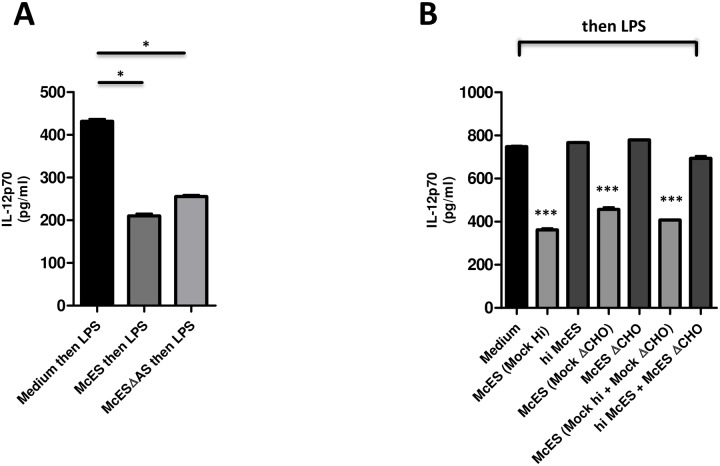
The BMDC-suppressive principle within McES is a glycoprotein. BMDCs were generated *in vitro* from C57BL/6 mice bone marrow cells and stimulated *in vitro* with 5 μg/ml of *M*. *corti* ES products (McES) for 24 h before restimulation with LPS. (A) IL-12p70 release following BMDC stimulation with McES or lipid-free (ammonium sulfate precipitated) McESΔAS then restimulation with LPS. (B) IL-12p70 release following BMDC stimulation with McES, heat-treated (denatured protein) HiMcES or carbohydrate-free McESΔCHO then restimulation with LPS. The data are representative of 2–3 independent experiments. p>0.05, NS; p<0.05, *; p<0.01, **; p<0.001, ***; p≤0.0001, ****.

To better understand the molecular nature of the immunomodulatory component within McES mixture, biochemical assays for the selective depletion of major classes of biomolecules were applied. To determine the role of free lipids, we comparatively tested the ability of McES and lipid-free McES or McESΔAS (i.e. ammonium sulphate precipitated as described in [27,28]) to impair BMDC activation. As shown in Fig 4A, similarly to McES, lipid-free ES significantly diminished the production of LPS-driven IL-12p70 by BMDCs indicating that parasite released lipids are not required for McES-mediated BMDC suppression.

Next, we appraised the role of parasite proteins contained in McES by comparative testing of heat-inactivated McES (hiMcES) and mock-treated McES (Mock hiMcES). As expected, pre-exposure to Mock hiMcES significantly impaired BMDC ability to release IL-12p70 in response to LPS (Fig 4B). In contrast, hiMcES completely failed to show such an ability (Fig 4B) indicating that parasite proteins are important for McES-driven BMDC suppression.

To determine the role of parasite-derived carbohydrates in the ability of McES to impair BMDC activation, we cleaved carbon bonds that bear hydroxyl groups using metaperiodate yielding carbohydrate-free McES (McESΔCHO) or performed a mock cleavage of McES using buffer without addition of metaperiodate (Mock ΔCHO). Predictably, Mock McESΔCHO impaired LPS-driven IL-12p70 release by BMDCs whereas McESΔCHO failed to show such an effect (Fig 4B). These results indicated that parasite carbohydrates are important for the suppressive effect of McES. Together, these findings demonstrated that both parasite proteins and carbohydrates are necessary for the immunosuppressive effect of McES.

Whether McES-driven immunosuppression requires a concerted action of a separate protein and carbohydrate entity or whether single or multiple entities combining carbohydrate and protein parts are responsible for the observed McES potency to suppress DC function remained unclear. To address this, we combined protein-depleted with carbohydrate-depleted McES and tested for their ability to impair LPS-driven BMDCs activation. As shown in Fig 4B, the mixture of protein-depleted McES (hiMcES) and carbohydrate-depleted McES (McESΔCHO) failed to inhibit LPS-driven IL-12p70 production by BMDCs whereas the mixture of mock controls (mock hiMcES and mock McESΔCHO) mediated this suppressive effect. These results indicated that one or several intact parasite glycoproteins, but not separate protein and carbohydrate entities, mediate(s) DC suppression by McES.

### McES but not larval homogenates impair BMDC activation

**Fig 5:**
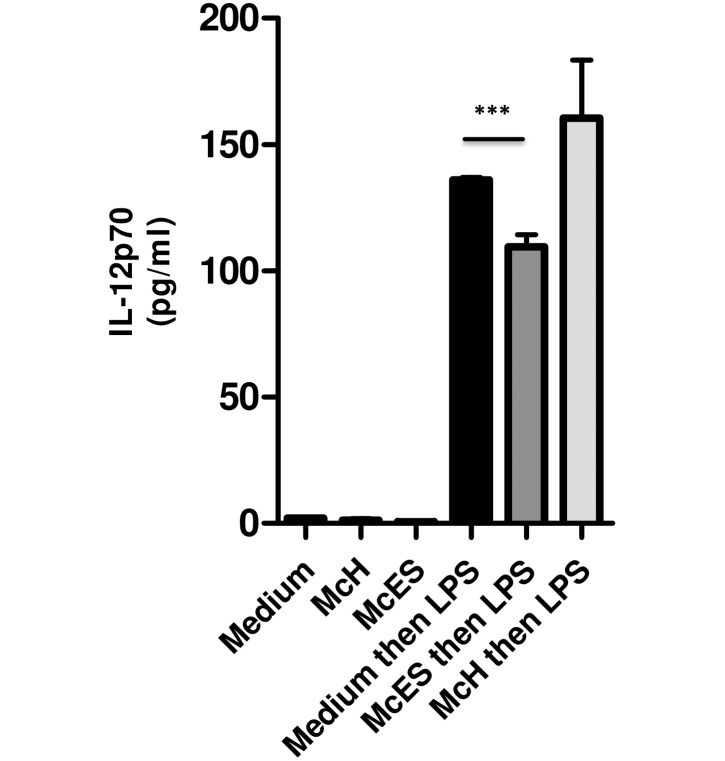
McES, but not McH impair TLR ligand-induced BMDC activation. BMDCs were generated *in vitro* from C57BL/6 mice bone marrow cells and stimulated *in vitro* with 5 μg/ml of *M*. *corti* ES products (McES) or larvae homogenates (McH) for 24 h. McH and McES–treated BMDCs were then restimulated with LPS for additional 24 h. IL-12p70 release following BMDC stimulation with McES or McH after 24 h or after restimulation with LPS at 48 h is shown. The data are representative of 2–3 independent experiments. p>0.05, NS; p<0.05, *; p<0.01, **; p<0.001, ***; p≤0.0001, ****.

To identify the distribution of the DC-suppressing protein(s) in *M*. *corti* tetrathyridia products, we comparatively evaluated the ability of *M*. *corti* homogenates (McH) and McES to impair LPS-driven IL-12p70 production by BMDCs (Fig 5). As expected, as little as 5 μg/ml of McES reduced LPS-driven IL-12p70 production by 50% in BMDC cultures whereas a similar amount of McH failed to show any suppressive effect on LPS-driven IL-12p70 release by BMDCs (Fig 5). Our data therefore suggested that the DC-suppressing glycoprotein(s) from *M*. *corti* tetrathyridia is/are specifically secreted by the larva and not that somatic products were leaking from the larval soma.

### Proteomic comparison of McH and McES

**Fig 6:**
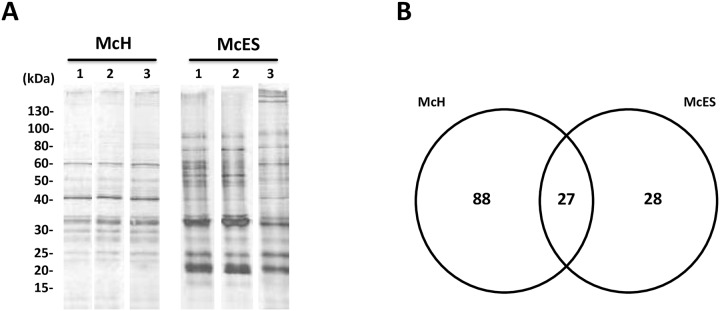
Comparison of McH and McES proteins. (A) One-dimensional SDS-PAGE of *M*. *corti* somatic homogenate (McH) and excretory-secretory products (McES). Three independents batches of McH and McES were compared by 1D SDS-PAGE; each lane was loaded with 0.5 μg of protein. Molecular weights markers are indicated on the left. (B) Venn diagram of total proteins from McH vs. McES enumerating specific and shared identities.

We next reasoned that the differential suppressive activity of McES when compared to McH might be the result of a differential expression of the immunomodulatory glycoprotein(s) in both parasite preparations. We therefore examined proteins differentially expressed between McH and McES to identify candidate DC suppressors. To this end, a 1D SDS-PAGE with three different batches of McH and McES was performed and proteins revealed by silver staining (Fig 6A). Visual inspection of the gels showed a general consistency in the protein composition and concentration across batches of each set of parasite products, although minor differences were evident (Fig 6A). Even though shared protein bands between McH and McES were visible, differentially represented protein bands were detected (Fig 6A). All protein bands were excised and processed for LC-MS/MS analysis to complement the visual comparison of McH and McES proteomes (S1–S3 Tables). We identified 115 *M*. *corti* proteins in McH and 55 *M*. *corti* proteins in McES (Fig 6B; S1–S3 Tables) thereby greatly extending the database of cestode proteomes beyond previously available information [18]. Of the 55 identified McES proteins, 27 (49%) were also detected in McH (Fig 6B, S3 Table) leaving 28 (51%) of the identified proteins exclusive to McES (Fig 6B, S1 Table). Notably, 88 (76.5%) of the 115 proteins identified in the McH were found to be exclusive to the parasite homogenates (Fig 6B, S2 Table). These results show that McES is markedly less complex than McH (in excess of 60 proteins) consistent with the observation that *M*. *corti* tetrathyridia differentially secrete a defined set of proteins (Fig 6B).

Among the 55 McES proteins identified were a selection of enzymes (i.e. proteases, phosphatases, enolases, lipases, aldolases, hydroxylases, fucosidases, isomerases, hydrolases, dehydrogenases and kinases), ion-binding proteins, protein transporters, fatty acid-binding proteins, conserved structural proteins (actins, heat shock protein, collagen and actin-binding proteins) and conserved regulatory proteins (ubiquitins, 14-3-3 proteins and endophilins). Protease inhibitors (cystatins, serpins) and annexins were also detected as were a high number of proteins with unknown functions with homologues in other cestodes (conserved cestode proteins) and novel proteins as yet unidentified in other helminths (S1 and S3 Tables).

All McES proteins were functionally annotated according to the Gene Ontology Consortium (http://geneontology.org/). GO terms analysis was performed to identify terms that were represented in the McES (S1 Fig). GO terms were assigned to the identified proteins on the basis of similarity using Blast2GO. In this analysis, ≥1 GO terms were assigned for 41 protein sequences (74.5%) of the total of McES product set. In total, 70 GO terms were returned (S1 Fig). These encompassed the three organizing categories of the GO database: biological process, molecular function and cellular component (S1 Fig). Only 12 cellular component ontologies were returned ranging from intracellular, membrane components to extracellular terms (S1 Fig). There were 40 biological process terms represented predominantly by terms for metabolic process, single organism process and cellular process (S1 Fig). There was an intermediate number of molecular function terms returned (18), representing a variety of terms with the most returned terms being ion binding, protein binding and hydrolase activity, respectively (S1 Fig).

Of the 55 proteins identified in the McES, 15 (27%) contained a predicted N-terminal signal peptide (S2 Fig). Nine (60%) of these classically secreted proteins did not correspond to any annotated gene in the NCBI database, and of these 5 (33% of classically secreted proteins) were novel proteins with no database match other than that of *M*. *corti* predicted genes (http://www.sanger.ac.uk/resources/downloads/helminths/; 01/29/2015 version). Of the identified 15 classically secreted proteins, 4 (27% of classically secreted proteins) matched predicted or hypothetical proteins of unknown function from other parasitic cestodes (S2 Fig). These results further illustrate the presence of a novel set of secreted proteins in McES.

**Fig 7:**
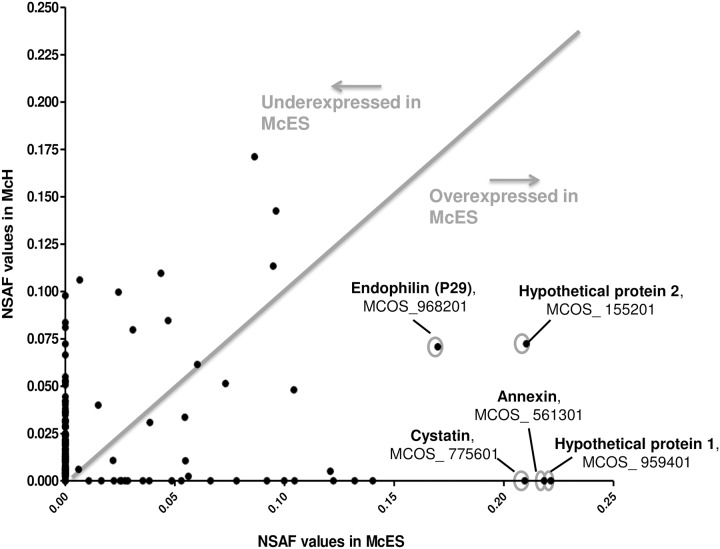
Relative abundance of proteins in McH and McES. Bivariant plot of NSAF values of proteins from McH and McES. Proteins over-represented in McES are highlighted.

We also compared the relative abundance of proteins in McES and McH by their NSAF values (normalized spectral abundance factors, [29]). We were able to infer that the relative concentration of a number of proteins differed substantially between McES and McH (S1–S3 Tables). To narrow down the database of most likely immunomodulatory proteins within McES, we searched for proteins with exclusive to over-representation in the immunomodulatory McES but absent or present in poorly detectable levels in the non-active McH (Fig 7). Specifically and by descending order of magnitude, the 5 most represented McES protein entities identified were: a novel hypothetical protein (MCOS_959401, exclusively detected in McES), an annexin homologue (Number 1, MCOS_561301, exclusively detected in McES), a hypothetical protein conserved among parasitic cestodes (Number 2, MCOS_155201, ~3-fold enrichment in McES), a cystatin (MCOS_775601, exclusively detected in McES) and an endophilin (MCOS_968201, 2.4-fold enrichment in McES) (S4 Table).

### Fractionation and proteomic analysis of the immunomodulatory fractions of McES

**Fig 8:**
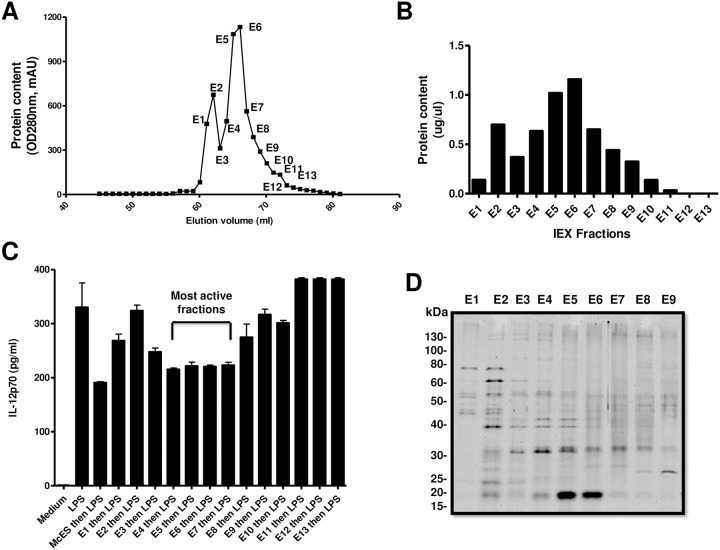
First line fractionation of McES by Ion Exchange Chromatography and Bio-assessment. McES dialysed against Tris-HCl was separated on an Ion exchange column at pH 6 using a linear salt (NaCl) gradient-based elution. (A) Protein elution profile of McES at 280 nm. (B) Protein concentration of eluted fractions of McES, as measured by Bicinchoninic acid assay. (C) LPS-driven IL-12p70 release following BMDC pre-exposure to 5 μg/ml of each McES fractions. Most active IEX fractions are indicated. (D) One-dimensional SDS-PAGE of 1 μg of the most represented Ion-exchange-chromatography-based McES fractions.

Column-based chromatographic analyses were used to further isolate the active fractions of McES. First, McES were fractionated by anion-exchange chromatography (IEX), dialyzed against PBS and tested for the immunosuppressive activity of the isolated fractions by IL-12p70 suppression (Fig 8). This first line of McES fractionation generated 13 detectable protein-containing fractions, as judged by the absorbance at 280nm (Fig 8A). The collected fractions were further dialyzed against PBS to minimize interference from the salty elution buffers on the downstream immunological assays. The protein concentrations in all fractions were determined (Fig 8B) tightly aligning to the previously obtained chromatogram (Fig 8A). To assess the immunomodulatory potency, IL-12p70 production by BMDCs pre-exposed to 5 μg/ml of each of the purified fractions before LPS stimulation was measured. As a positive control of DC-suppressing fraction, 5μg/ml of total McES, resuspended in elution buffer and further dialyzed against PBS was used. As expected, exposure of BMDCs to total McES reduced the LPS-driven production of IL-12p70 by close to 50% (Fig 8C). The activity was different across McES fractions. Whereas fractions E1, E3, E4, E5, E6 and E7 displayed a considerable ability to impair LPS-driven BMDC production of IL-12p70, fractions E2, E8, E9, E10, E11, E12 and E13 failed to do so (Fig 8C).

To have a visual appraisal of the protein composition of the various McES fractions, we performed a one-dimensional SDS-PAGE of 11 μg from each fraction (Fig 8D). A complex but rather dissimilar banding pattern was apparent in the major protein-containing fractions of McES. We failed to clearly detect a differential banding pattern between active and non-active fractions as both group of fractions revealed protein bands spanning the entire MW range (<15 to >130kDa). Notably, in the minimally active fraction E1 (around 12% inhibition), only proteins of MW higher than 40kDa were visible.

**Fig 9:**
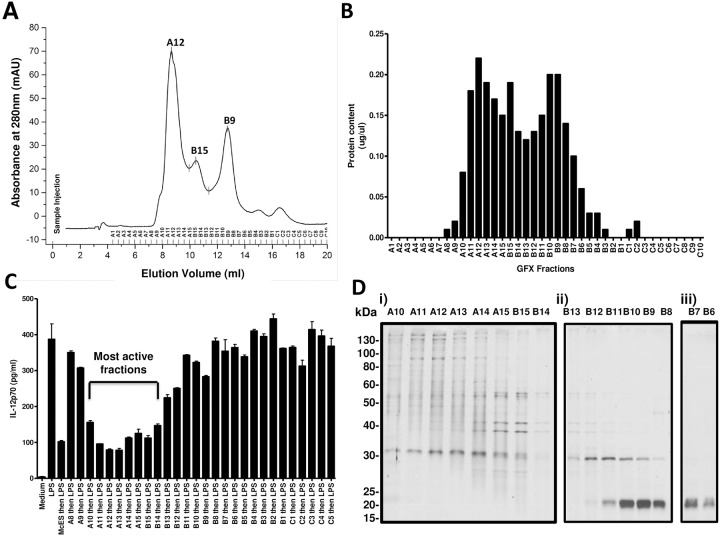
Second line fractionation of McES by Gel Filtration Chromatography and Bio-assessment. Fractions of McES isolated by anion exchange chromatography that display a suppressive potential on LPS-driven BMDC activation were pooled together and loaded on a Superdex 200 10/300 column (GE Healthcare) for further fractionation by gel filtration chromatography. The column was equilibrated with 2 column volumes of PBS (pH 7) before elution with PBS. (A) Superdex 200 chromatogram of McES fractions. The fractions were monitored by recording the absorbance at 280 nm for protein content. (B) Protein concentration of eluted McES fractions, as measured by Bicinchoninic acid assay. (C) LPS-driven IL-12p70 release following BMDC pre-exposure to 5 μg/ml of each McES fractions. Most active GFX fractions are indicated. (D) One-dimensional SDS-PAGE of 1 μg of the most represented gel-filtration-based McES fractions. Proteins are visually clustered on the gel into 3 groups of molecular weight i.e. group i) 25->130kda, group ii) <15–40 kDa and group iii) < 15kDa.

To further characterize the protein entity(ies) that mediate the immunosuppressive potential of McES, fractions E4, E5, E6 and E7, representing the most active fractions (Fig 8C) with the highest protein content (Fig 8B) were pooled and the mixture was subjected to gel filtration chromatography (GFX, Fig 9). This second line of McES fractionation generated 3 detectable protein-containing peaks as judged by the absorbance at 280 nm (Fig 9A). These peaks were further subdivided under refined elution profile of 40 fractions (Fig 9A) to increase the resolution of our analyses. The protein concentration in all fractions was determined (Fig 9B) tightly reflecting chromatogram (Fig 9A). To assess the immunomodulatory potency, IL-12p70 production by BMDCs pre-exposed to 5 μg/ml of each of the fractions before LPS stimulation was measured. As a positive control, 5 μg/ml of active McES fractions from IEX were used. As expected, exposure of BMDCs to the pool of active McES fractions after IEX reduced the LPS-driven production of IL-12p70 by close to 75% (Fig 9C). The activity was different across McES fractions after GF revealing A10, A11, A12, A13, A14, A15, B15, B14 as the most active fractions mediating at least a 50% reduction of LPS-driven IL-12p70 release by BMDCs (Fig 9C). Fractions B11, B10, B9, B8, B7, B6, B5, B4, B3, B2, B1, C1, C2, C3, C4, and C5 were poorly to non-active mediating less than 50% of reduction of LPS-driven IL-12p70 release by BMDCs (Fig 9C). To have a visual appraisal of the protein composition of the various McES fractions, we performed a one-dimensional SDS-PAGE of each fraction (Fig 9D). A clear clustering of the protein banding pattern along three definable MW groups was observed for the major protein-containing fractions (Fig 9D). These groups of proteins were arbitrarily defined as group i (>130kDa to 25kDa), group ii (60kDa to 20kDa) and group iii (< 20kDa). Active fractions were principally found within the MW group (i) suggesting that the active principle within McES might be of a MW >20kDa since fractions from group ii and iii showed minimal to no activity (Fig 9C and 9D). Taken together, our bio-activity based fractionation analyses suggest that (a) protein(s) of MW higher than 20 kDa might mediate the immunosuppressive activity of McES.

Having now isolated refined fractions of McES with differential DC-suppressing activity, we reasoned that a comparative proteomic analysis of active and non-active McES fractions could provide us with a list of candidate DC-suppressing proteins preferentially represented in active fractions. To address this, fractions A14, A15 and B15 as the most active fractions (Fig 9C) with highest protein content (Fig 9D) and fractions B6, B7, B8 and B9 as the non-active fractions (Fig 9C) with high protein content (Fig 9D) were individually lyophilized and analyzed by mass spectrometry for their protein composition.

**Fig 10:**
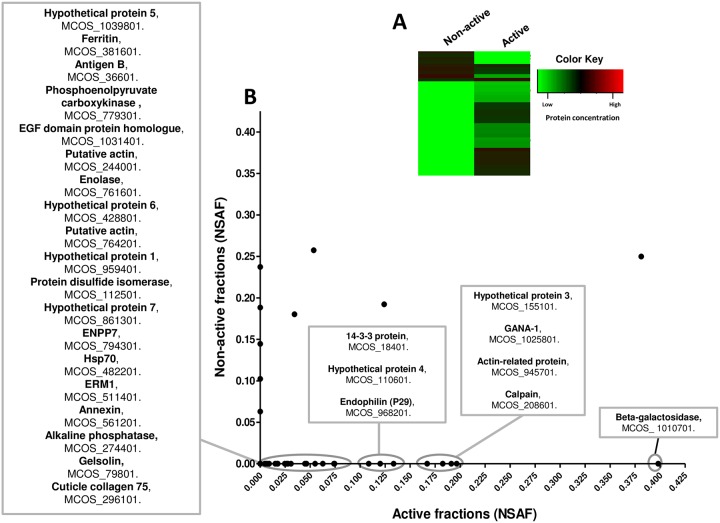
Mass spectrometric-based comparative proteomic analysis of active vs. non-active fractions of McES. (A) Heat map showing the mean relative abundance of the indicated proteins identified in all McES fractions investigated. (B) Bivariant plot of NSAF values of proteins from active and non-active McES fractions. McES fractions after gel filtration chromatography were separated into active vs. non active depending on their ability to impair LPS-driven BMDC activation. 3–4 fractions from the most active and non-active fractions were lyophilized and analysed by LC-MS/MS. Proteins exclusively present in active fractions are displayed in grey boxes in a descending order of NSAF values. For the active or non-active group of fractions, the computed NSAF value of a given protein is the mean of the individual NSAF values of this protein in the 3–4 different fractions analyzed.

**Fig 11:**
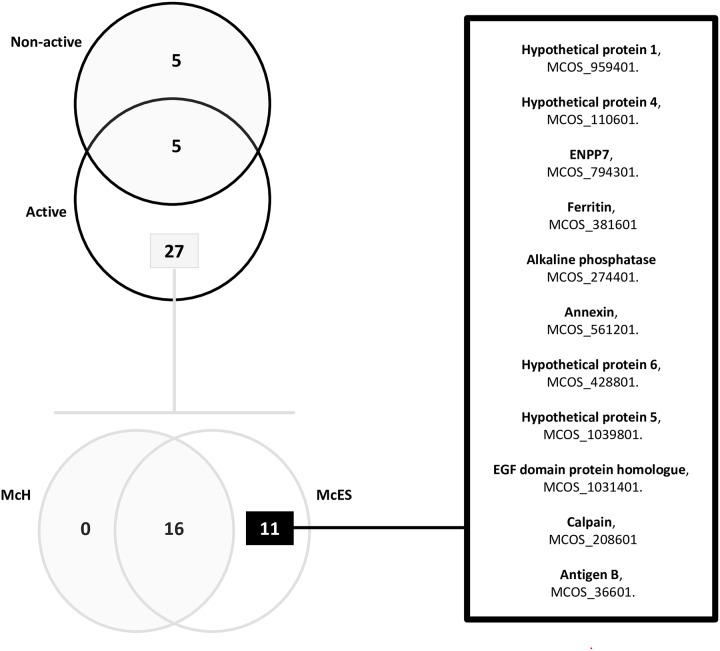
Distribution profile of proteins identified in McES fractions. Venn diagram of all proteins detected in McES fractions (Upper left) of which proteins exclusively present in McES active fractions are further distributed according to their detection in McH and McES (Lower left). A refined list of proteins exclusive to active preparations (immunomodulatory fractions and McES) of *M*. *corti* products is provided.

Overall, 37 different proteins were detected in the analyzed McES fractions (S5 Table). To assess the differential profile of active and non-active fractions of McES, the relative abundance of each protein defined by the NSAF values (normalized spectral abundance factors, [29]), was plotted revealing a mutually exclusive distribution of the majority of proteins to either active or non-active McES fractions (Fig 10). Close to 77% (27/37) of all identified proteins were exclusive to the active fractions (Fig 11) of which 40% (11/27), not detectable in the parasite somatic extracts, were associable to McES immunomodulatory potential i.e. never detected in non-active parasite products and frequently/always present in active parasite products (Fig 11, S6 Table). Those are by descending order of representation in *M*. *corti* ES products, a hypothetical protein (number 1, MCOS_959401), another hypothetical protein (number 4, MCOS_110601), an ectonucleotide pyrophosphatase/phosphodiesterase family member 7 (ENPP7, MCOS_794301), a ferritin (MCOS_381601), an alkaline phosphatase (MCOS_274401), an annexin (MCOS_561201), a hypothetical protein (Number 6, MCOS_428801), another hypothetical protein (number 5, MCOS_1039801), an epidermal growth factor (EGF)-domain protein homologue (MCOS_1031401), a calpain (MCOS_208601) and the cestode antigen B (MCOS_36601).

## Discussion

The infectious cestode larvae intimately dwell within their mammalian host organs, mitigating the host immune response [1–3]. Their longevity in mammalian hosts raises a growing interest in the molecular basis of host immunomodulation by these parasitic larvae [30]. Generally, immunosuppression by helminths results from prior interaction with innate immune cells–such as DCs–and relies on viable parasites and their released products [13,31]. However, much on the mechanisms of host immunomodulation by released products of cestode larvae remains to be elucidated.

In the current study, we have characterized the immunomodulatory potential of ES products from the tissue-dwelling metacestode larva of *M*. *corti* at the level of IL-12p70 production by BMDCs. Our results show that secreted glycoproteins from *M*. *corti* tetrathyridia impaired the IL-12p70 secretion of BMDCs in response to a wide-range of pro-inflammatory or microbial stimuli. We performed an extensive proteomic analysis of these products and provided a comprehensive library of *M*. *corti* tetrathyridium-derived proteins that might suppress DC functions.

DCs play a sentinel role in the sensing and recognition of invading pathogens [32]. These cells initiate immune response through several signals: (i) antigen presentation via MHC-II molecules, (ii) the expression of co-stimulatory molecules such as CD86 and (iii) cytokine production [32–34]. Beside quantitative and qualitative importance of each signal, IL-12 is a key inflammatory cytokine for the development of a parasite-limiting Th1 immune response [32–34]. By injecting *M*.*corti* tetrathyridia into the peritoneum of mice, we first demonstrated an early role for DCs in the interactions between the mammalian host and *M*. *corti* tetrathyridia in the course of an infection. A central role for live larvae-released products (ES products) in the interaction of *M*. *corti* with the host immune system was uncovered here as live but not dead larvae continuously recruited DCs to the peritonea of injected mice throughout the first 7 days post-infection. Our subsequent observation of a persistently heightened recruitment of host immune cells within the peritonea of animals injected with live, but not dead *M*. *corti* tetrathyridia further supported the critical role of *M*. *corti* tetrathyridia ES products in facilitating the parasite persistence *in vivo*. This is consistent with an increasingly appraised role of helminth ES products as the most physiologically relevant parasite-derived products that mediate the fine-tuning of hosts by parasites [11,35] Indeed, we reported earlier that ES products from *M*. *corti* tetrathyridia inhibit DC activation [22] similar to ES products identified from other metacestodes [7,8,13,14,36–38]. As oppose to our previous observations on ES products from *Echinococcus multilocularis* larvae [7], we failed to detect any DC-killing activity in *M*. *corti* ES preparations. This demonstrates that although phenotypically similar, the mechanisms of host immunomodulation by products of parasitic cestodes might not be redundant from one species to another. As an example, the most exposed structure of the closely related *Echinococcus spp*, the laminated layer rather promotes DC maturation [39]. Contrarily in our study, LPS-driven DC maturation was not affected by *M*. *corti* ES products or homogenates and this was also inconsistent with the widely reported ability of secretions from tissue-dwelling larvae of other parasitic cestodes like *E*. *multilocularis* [7], *E*. *granulosus* [38] or *T*. *crassiceps* [8,14] to impair LPS-driven dendritic cell maturation.

Our study shows that McES not only limited DC activation independently of the timing for stimulation after exposure to the parasite products as they also suppressed IL-12p70 release by BMDCs that had already been activated with LPS. The ability to refrain BMDCs from LPS-driven activation by simultaneous exposure to released products is a rather common ability of ES products of parasitic helminths [31], but the impairment by *M*. *corti* tetrathyridia ES products of the immune effector functions of DCs that had already been activated is interesting. In fact, such products that can refrain IL-12 production by inflammatory DCs are clearly encouraging in the quest for novel and more effective approaches to counteract IL-12-dependent inflammatory diseases like sepsis [40]. Moreover, we also observed that McES mediated a general DC unresponsiveness to several TLR ligands and non-TLR ligands, which activate pathways that are instrumental for the pathogenesis of sepsis [41] further supporting the robust anti-inflammatory potential of McES. Such a potential of McES could both serve the silent establishment of the parasite in the course of an infection with *M*. *corti* tetrathyridia but could equally help preserve the host from tissue destruction by a frustrated and uncontrolled anti-tetrathyridia immune response.

Our work also uncovered a glycoprotein nature of the mediator(s) of the DC-suppressing effect in McES. This is not uncommon since glycoproteins have already been widely reported to play a crucial role in the priming of the host immune cells by products of cestode larvae [13,42]. The suppressive activity of McES could not be detected in the somatic products of *M*. *corti* tetrathyridia, a quite intuitive observation given the large representation of glycoproteins among helminth-released products when compared to somatic products [11,17,19–21,30]. Proteomic analyses of *M*. *corti* tetrathyridia products enabled us to identify 143 proteins of which 55 were detected in the ES products and 88 in the somatic products. Comparisons between the two sets of proteins indicated a considerable lack of overlap supporting the purity of our McES preparations and excluding the leakage of somatic antigens from degenerating tetrathyridia as the primary source of the identified McES proteins. Moreover, since only 23% of the proteins detected in the McH could also be found in the McES, a selective and regulated transit of proteins from the parasite soma to the exterior milieu under our serum-free cultivation conditions is also strongly supported. Additionally, gene ontology analyses of the proteins identified within the McES could only ascribe stress response to 2 of the detected proteins (out of 41 proteins annotated) further indicating that ES products collected under our culture conditions were not from metabolically impaired/stressed larvae. Since *in vitro* generated ES products of *M*. *corti* can be recognized by IgG antibodies from chronically infected mice [43] and some composing entities of these products can be detected in the serum of infected mammals [19,44], we expect that our McES collected *in vitro* reflects an analogical release by *M*. *corti* tetrathyridia *in vivo*.

In this regard, it is noteworthy to point out the abundance in our McES of previously characterized immunomodulatory proteins. Many of them perform biological function necessary for the parasite survival. For example, proteases known to participate in the establishment and maintenance of infections [45]. Similarly, we detected fatty acid binding proteins which are involved in the transport of hydrophobic molecules, generally used as substrates for energy metabolism and signaling and capable of inducing alternative activation of macrophages (AAM) [46]. Another group of molecules, the glutathion-transferases, important in the detoxification of reactive oxygen species released from the host cell and in the inhibition of inflammatory responses were also identified in our McES [47]. Overall, *M*. *corti* tetrathyridia aided by their ES products might support energy supply, protect from the hardship of the host immune effector responses and help tame anti-parasitic host immune responses, all supportive of the parasite survival and the progression of the infection.

Our findings on the comparative proteomic profiling of McH and McES then further revealed a set of proteins that were highly or exclusively present in the DC-suppressing McES as compared to the non-active McH. Intriguingly, 2 out of the 5 most represented of these proteins (hypothetical protein 1, MCOS_959401 and hypothetical protein 2, MCOS_155201) were of unknown function and harbored secretory motif suggesting their active release by *M*. *corti* tetrathyridia at the host parasite interface. Such proteins with unknown functions, if proven to be immunosuppressive, might provide novel insights on how to regulate DC responses but more investigations are clearly required at this level. A cysteine-rich secretory protein-3 (CRISP-3) containing a CAP domain was also exclusively detected in McES. CRISP-3 shares similarities with Venom Allergen-Like (VAL) proteins, which are dominant in ES products of nematodes where they represent interesting candidates for vaccine development [16]. Whether this host-protective function is relevant in *M*. *corti* tetrathyridia-mediated infections still remains to be determined.

The most represented proteins in McES include annexin, cystatin and endophilin. Cestode annexin hold the highest homology with annexin A13, a member of the annexin family that has not been functionally characterized yet [48]. Although present in the genome of most classes of parasitic helminths [49], cystatins from nematodes are the most functionally characterized helminth cystatins shown to inhibit, among others, proteases involved in antigen processing and presentation, which diminishes T cell responses [49,50]. *M*. *corti* endophilin is considerably similar to *Echinococcus* spp. P29 proteins, which were shown to be highly efficient host protective antigens when used as vaccines [51,52]. A similar role for *M*. *corti* endophilin might therefore be supposed but would require experimental validation.

The overall goal in the present study was to identify the *M*. *corti*-derived immunomodulatory glycoprotein(s) which suppress IL-12 pro-inflammatory cytokine release by DCs. Sequential bioassay-guided chromatographic fractionation of McES helped pin down a list of *M*. *corti* proteins exclusively present in the McES fractions that were suppressive to BMDCs. Moreover, a visual analysis of one-dimensional protein gels of McES fractions further helped narrow down the likely DC-suppressing factor(s) to a molecular weight higher than 20kDa. Therefore, focusing on the differential proteome between active vs. non-active McES fractions and eliminating candidates detected in the non-active parasite somatic extracts and keeping in mind the glycosylated nature of the DC-suppressive principle(s), we identified 11 candidates DC-suppressing proteins. Of these, several appear not to match any protein of known function further indicating the potential of the current library of proteins in uncovering novel scheme(s) of metacestode interactions with their mammalian hosts. The functional characterization of these factors is currently underway and will be greatly facilitated by the commendable recent efforts in the sequencing and annotation of the genome of the major parasitic cestodes [53,54]

In conclusion, we have dissected the ES products of the tissue-dwelling tetrathyridium of the model cestode *M*. *corti*. Importantly, the extension of these findings to more clinically/economically relevant metacestodes and the potential of the identified proteins as anti-cestode vaccines and/or controllers of unwanted host immune responses altogether re-emphasize the value of the library of candidates provided in the present work.

## Materials and Methods

### Ethics statement

Animals handling, care and all experiments were carried out in compliance with Slovakian (Law No. 23/2009) regulations on the protection of animals. All *in vivo* experiments were performed at Institute of Parasitology, Slovak Academy of Sciences (Slovakia) following ethic approval of the protocol 1359/14-221a under the law 39/2007 as amended by the local ethics committee of the State Veterinary Administration of the Slovak Republic in agreement with the Slovak Republic Government regulation number 377/2012.

### Mice

ICR and BALB/c mice were bred and housed at the animal facilities of the Institute of Parasitology, Slovak Academy of Sciences (Slovakia) under specific pathogen-free conditions. C57BL/6 mice were purchased from Charles River/Wiga (Sulzfeld, Germany) and bred within the animal facility of the Institute of Virology and Immunobiology, University of Würzburg (Germany) under specific pathogen-free conditions. Mice were used at the age of 6–10 weeks.

### Parasites, ES products and somatic protein homogenates

*M*. *corti* tetrathyridia were maintained in experimental hosts and cultivated essentially as described by Vendelova *et al*. [22]. Briefly, tetrathyridia were maintained in ICR mice (6–8 week old) by the serial passage upon oral infection of larvae obtained from the peritoneal cavity of mouse with chronic infection. Host cells were removed from parasite material by axenic cultivation as previously described [22]. Tetrathyridia were maintained for 14 days in serum-free tissue culture medium (DMEM+Glutamax, GIBCO) containing antibiotics (100 U/ml of penicillin, 100 μg/ml of streptomycin) (Biochrom, Berlin, Germany), 20 μg/ml Levofloxacin (Tavanic, Sanofi-Aventis) and 50 μM 2-mercaptoethanol (Merck, Darmstadt, Germany). Larvae viability was assessed by motility. Culture medium conditioned with parasite products from *M*. *corti* cultures which remained viable throughout the observed period was collected every 24 h and processed as previously defined [22]. Briefly, for a preparation, supernatants were pooled together, sterile-filtered through 0.22 μm pore-size filter (Minisart Sartorius, Gottingen, Germany), concentrated 30 times and the buffer exchanged to phosphate-buffered saline (PBS) (Sigma, St. Louis, USA) using a 3 kDa concentrating column (Merck Millipore, Tullagreen, Carrigtwohill Co., Cork, Ireland).

To obtain the somatic homogenate (McH), *M*. *corti* tetrathyridia from *in vitro* cultures were extensively washed and mechanically squeezed with glass tissue grinder in cold PBS. The supernatant from homogenized larvae was collected and sterile-filtered through 0.22 μm pore-size filter. All procedures were performed under strict aseptic conditions. Protein concentration was determined using BCA Protein Assay Kit (ThermoFisher Scientific) and samples were stored at -80°C until required. Independent aliquots (from different parasite isolates) of McH and McES were lyophilized for LC-MS/MS analysis.

### Treatment of ES products

The involvement of intact protein in immunomodulation was investigated upon heat-inactivation (hiMcES) in water bath at 100°C for 15 min. Mock-treated ES were kept 15 min at room temperature (Mock hi). To test the carbohydrates involvement, sodium metaperiodate-mediated modification of glycan moieties was performed. Briefly, 0.5 mg/ml of ES mixture was treated with 100 mM (vol/vol) of sodium acetate (pH 5.5) at room temperature. The tube content was divided to obtain test sample with addition of sodium metaperiodate (10mM) in sodium acetate buffer (McESΔCHO) or control mock-treated ES products (Mock ΔCHO) treated with the equivalent amount of sodium acetate buffer without sodium metaperiodate. The samples were incubated in the dark at room temperature with gentle shaking for 1h. Desalting and buffer exchange to PBS was accomplished using the Amicon Ultra-0.5 (3K MWCO; Merck Millipore) as per manufacturer instructions. To selectively precipitate proteins, ES products were saturated with ammonium sulfate up to a concentration of 80% (McESΔCHO). The precipitated proteins were obtained by centrifugation (6500g, 20 min) and dissolved in 100μl PBS and buffer-exchanged using Amicon-Ultra 0.5 (3K MWCO; Merck Millipore) as per manufacturer instructions.

### Experimental infections

Axenic *M*. *corti* tetrathyridia were washed 3 times and 60 larvae in 1 ml PBS were injected i.p. in Balb/c mice. Control mice received 60 dead larvae (heat killed by treatment at 100°C for 15 min) or 1 ml of PBS as a mock control. At day 3 and 7 p.i., mice were sacrificed by CO2 asphyxiation and peritoneal exudate cells were collected by flushing the peritonea with 5 ml of complete medium i.e. RPMI 1640 (Biochrom, Berlin, Germany) supplemented with 10% heat-inactivated fetal calf serum (Biochrom, Berlin, Germany), 100 U/mL of penicillin (Biochrom, Berlin, Germany), 100 μg/mL of streptomycin (Biochrom, Berlin, Germany), 2 mM L-glutamine (Sigma, St. Louis, USA) and 50 μM 2-mercaptoethanol (Merck, Darmstadt, Germany). The suspensions of peritoneal cells were sieved through a 40 μM nylon filters (BD Biosciences, Durham, NC, USA). Red blood cells were lysed using 1.4% NH_4_Cl for 5 min at 37°C and then washed with the complete RPMI medium. Viable cells were counted using a Neubauer chamber by trypan blue exclusion.

### Generation of bone marrow-derived DCs (BMDCs)

BMDCs were generated from mice bone marrow precursors of C57BL/6 mice by GM-CSF as previously described [55]. Briefly, 2–3 x 10^6^ precursor cells were cultured in complete RPMI medium supplemented with GM-CSF at 37°C, 5% CO2. Cultures were fed with GM-CSF on days 3 and 6. On day 8, non-adherent and semi-adherent cells representing differentiated DCs (80–90% CD11c+) were harvested and washed in complete medium prior to *in vitro* stimulation assays.

### *In vitro* stimulation of BMDCs with *M*. *corti* ES products

1 x 10^6^ BMDCs were plated in 24 well-plates (Nunc, Roskilde, Denmark) in complete RPMI medium. 5, 20 or 50 axenically maintained larvae were washed thrice and added directly into DC cultures. In another series of experiments, larvae were separated from BMDCs using trans-well inserts (0.4 μm, BD Falcon). Alternatively, larva ES products were used instead of whole larvae. Lipopolysacharide (LPS; 0.1 μg/ml, E. coli 0127:B8) was used as a positive control for DC activation. After 24h of DC stimulation at 37°C under 5% CO2, supernatants were collected for cytokine detection and cells were stained for flow cytometric analysis. For re-stimulation experiments, larvae were removed 24 h post stimulation and BMDCs were further stimulated with LPS (0.1 μg/ml, *E*. *coli* 0127:B8) for an additional 24 h. In some experiments, BMDCs were treated with different doses (0.5 μg/ml up to 50 μg/ml) of *M*. *corti* ES products or McH prior to, at the same time with, or 18 h after LPS stimulation. In several experiments different stimuli were used instead of LPS for restimulation i.e.: Zymosan A (*Saccharomyces cerevisiae*; 50 μg/ml, Sigma), Curdlan (50 μg/ml, Wako), Lipotechoic acid (LTA, 10 μg/ml, InvivoGen) or phorbol 12-myristate 13-acetate (PMA; 0.5 μg/ml, Sigma). Control BMDC wells were treated in a similar way without exposure to parasite larva samples.

### Flow cytometric analysis

Expression of cell surface markers on BMDCs was measured using anti-mouse fluorochrome-conjugated antibodies specific for CD11c lineage marker (clone N418; PE, APC, Pacific blue, FITC; eBioscience), MHC-II (M5/114.15.2; I-A/I-E; PE, Alexa fluor700; eBioscience) and CD86 (clone GL1; FITC, PE; eBioscience). For staining, cells were incubated with anti-CD16/CD32, stained with CD11c and thereafter washed in FACS buffer (1x PBS supplemented with 0.1% BSA and 0.1% NaN3). To exclude cell debris, DRAQ5 (Abcam) (5 μM) was added. *In vitro*-generated BMDCs were stained for 30 min with a cocktail of CD11c, MHC-II and CD86 antibodies and washed in FACS buffer. To analyze the cell death, BMDCs were stained for 20 min with a staining mix composed of 1x annexin-V binding buffer (BD Pharmingen) containing annexin-V (roman 5; FITC, BD Pharmingen) and Propidium Iodide (PI; BD Pharmingen). Cells were acquired on a FACSCalibur (Beckton Dickinson) or BD LSRII equipped with DIVA software (BD Biosciences, San Jose, USA). Analyses were done on FlowJo software (Tree Star, USA).

### Measurement of cytokine release by BMDCs

Culture supernatants were harvested and stored at -20°C. The production of IL-6, IL-10, IL-12p70 and IL-23 was assessed using sandwich ELISA (OptEIA kits, BD Biosciences or Ready-SET-Go, eBioscience) according to the manufacturer´s instructions. The kit detection limits were 15 pg/ml for IL-12p70 and IL-23 and 19 pg/ml for IL-10 and IL-6.

### Anion exchange chromatography (IEX)

The concentrated ES products (resuspended in PBS) were dialyzed twice against 20 mM Tris-HCl (pH 8) using Thermo Scientific Slide-A-Lyzer G2 Dialysis Cassettes (3K MWCO; Life Technologies). The dialysate was centrifuged at 30 000 g for 20 min at 4°C to remove precipitates. Approximately 10 mg of ES products were subsequently loaded onto an anion-exchange HiTrap-Q HP column (GE Healthcare), which was connected, to an ÄKTA Purifier FPLC system (GE Healthcare) and equilibrated with a low-salt buffer. To determine the best conditions for separation of the ES products, pH scounting was performed using a triple pKa buffer/HCl system (CIEX: 30 mM di-sodium phosphate, 30 mM sodium formate and 60 mM sodium acetate; IEX: 50 mM 1-methyl-piperazine, 50 mM BisTris base, and 25 mM Tris-base) as suggested by GE Healthcare and using a 1 ml HiTrap Q/S ion exchange column for separation in analytical scale. From these ion exchange runs, pH 6 provided the best resolution and was adopted for subsequent preparative IEX. Preparative IEX of the dialysate of McES was performed at pH 6 using the triple IEX pKa buffer/HCl system. Practically, before loading the sample, the 1 ml HiTrap Q column was equilibrated with 15 column volume 20 mM MES buffer (pH 6). Fractionated elution of bound ES proteins/components was performed employing a linear gradient 0 to 1 M NaCl (25 column volumes). Loading and elution was done using a flow rate of 1 mL/min. Protein elution was monitored by measuring the absorption at 280 nm, and fractions of 1ml each were collected. The obtained fractions were ultra-dialyzed extensively against PBS using Amicon-Ultra 0.5 (3K MWCO; Merck Millipore) before protein quantification by Bicinchoninic acid assay and functional assessment in the *in vitro* DC stimulation assay.

### Size exclusion chromatography

IEX fractions of highest DC-suppressive activity were pooled for Gel filtration chromatographic fractionation. The gel filtration was performed on an Äkta Explorer machine (GE Healthcare) using a Superdex 200 10/300 column (GE Healthcare) equilibrated with 2 column volumes PBS (pH 7). Approximately 500 μl of 2.5 mg/ml protein solution was applied. Loading and elution was done in the same buffer using a flow rate of 0.4 ml/min. Protein elution was monitored by measuring the absorption at 280 nm and 400 μl fractions were collected. The eluted fractions were probed by Bicinchoninic acid assay for protein quantification before testing in the *in vitro* DC stimulation assay. The tested fractions were analyzed by SDS-PAGE. Immunosuppressive fractions (3) and non-active fractions (4) were lyophilized and the protein composition analyzed by LC-MS/MS.

### Sample preparation for mass spectrometry

Protein samples were diluted in denaturing buffer (25 mM NH_4_HCO_3_/8 M urea, pH 8.0), reduced by adding DTT (1 μg/50 μg protein), and carboxyamidomethylated with iodoacetamide (5 μg/50 μg protein). Samples were then diluted to 1 M urea with 25 mM NH_4_HCO_3_ (pH 8.0), and trypsin (Trypsin Gold, Mass Spectrometry Grade, Promega) was added at a ratio of 1 μg/ 100 μg protein. After digestion for 4 h at 37°C, an additional aliquot of enzyme was added, and samples were further incubated for 16–20 h at 37°C. The resulting peptides were desalted using OASISs HLB Cartridge (Waters, USA) and lyophilized.

### LC–MS/MS data analysis

Peptides were analyzed using a Q-Tof Premier API mass spectrometer (MicroMass/Waters), attached to a nanoACQUITY ultra performance liquid chromatography (UPLC) system (Waters). Ten micrograms of each peptide sample were injected in an analytic ACQUITY UPLC peptide BEH C18 nanoACQUITY column (130 Å, 1.7 μm, 100 μm ×100 mm) with a 2–90% acetonitrile gradient in 0.1% formic acid, at a 200 nL/min flow rate, for 45 or 60 min, for ES products samples or tetrathyridium somatic products sample, respectively. An ACQUITY UPLC Symmetry C18 nanoACQUITY trap column (100 Å, 5 μm, 180 μm × 20 mm) was used for sample desalting at a flow rate of 5 μl/min over 2 min. The MS spectra between m/z 100 to 2000 Da were recorded, with 1-second search time spaced by 0.1-second interval. The MS/MS spectra were acquired on m/z 100–2000 Da mass range with the same search time and interval mentioned for the MS mode, using the MassLynx software system (Waters). The samples were analyzed at data dependent acquisition mode, in which every full MS mode run was followed by three consecutive MS/MS runs of the three most intense multiple charged ions with a count higher than the threshold (30 counts/s). The collision energy values necessary for the peptide fragmentation were adjusted according to the +2, +3 and +4 ion charges recognition files available by the MassLynx system. The raw MS/MS data were processed using the Mascot Distiller v. 2.2.1 (Matrix Science, Boston, MA, USA) to generate the *.mgf peak list files. Each sample was independently analyzed two or three times (as indicated) by LC-MS/MS (technical replicates).

### Database searching and bioinformatics

The MS/MS peak list data files were run through the Mascot ion search engine version 2.3.0, using carbamidomethylation of cysteine as a fixed modification (monoisotopic mass 57.0215 Da), methionine oxidation as a variable modification (monoisotopic mass 15.9949 Da), and a peptide and MS/MS fragment ion mass tolerance of 0.1 Da. Other parameters were set to include up to one missed cleavage, and the Mascot automatic decoy database search was selected. All protein searches were performed using the deduced amino acid sequences from the *M*. *corti* genome, available at ftp://ftp.sanger.ac.uk/pub/pathogens/bh4/ (version 29/01/15 09:48:00).

The *.dat files (obtained by Mascot platform) were merged (the LC–MS/MS technical replicates) and processed by ScaffoldQ+ version 4.4.1.1 (Proteome Software, Portland, OR, USA) as follows. Mascot ion scores of 30 or higher (for +2, +3 and +4 charges), a minimum of two identified peptides, 90% peptide identification probability (using the Scaffold Local FDR algorithm), and 99% protein identification probability were required to improve the reliability of protein identifications, resulting in a calculated FDR of <1%. The normalized spectral abundance factor (NSAF) [56] was calculated for each protein, and quantitative differences were statistically analyzed by a t-test using Scaffold Q+ version 4.4.1.1. Differences with p values lower than 0.05 were considered statistically significant. Differential proteins from t-test were submitted to hierarchical clustering analysis in Perseus software package (version 3.15).

Gene ontology (GO) functional classification of McES proteins was performed using Blast2GO [57], in which a BLASTP search using NCBI non-redundant protein database with a cut-off of 30 for homology annotation was applied. McES proteins were also investigated for the presence of signal peptide using SignalP 4.1 [58], and presence of non-classical signals using SecretomeP 2.0 [59]. A protein was considered to contain a signal peptide if the D-score was >0.5 and to be non-classically secreted if the NN score was higher than 0.6 (unless a signal peptide has been already predicted to a given protein).

### Statistical analyses

All results were expressed as mean ± standard deviation (SD). Differences observed were analyzed with nonparametric test that does not assume normality of the measurements. When only two groups were compared, a Mann-Whitney test was used. When 3 groups or more were compared, Kluskal-Wallis with Dunn post hoc comparison was used. Values of p<0.05 were considered statistically significant. Statistical analyses were performed with GraphPad Prism 6.00 for Windows (GraphPad Prism Software).

### Accession numbers

Most of the reported sequences are not yet available on publicly stable and available databases but can be retrieved from the “50 helminth genomes database” of the Wellcome Trust Sanger Institute at http://www.sanger.ac.uk/resources/downloads/helminths/

## Supporting Information

S1 Fig Gene ontology (GO) classification of proteins identified in McES. BLASTP searches were performed using Blast2GO against the NCBInr database. Most abundant GO terms for biological processes (A), molecular function (B) and cellular component (C) are shown.
(TIF)

S2 Fig Likely secretory pathways and novelty of identified McES genes with signal peptides. (A) Prediction of secretory pathways of proteins detected in ES products from *M*. *corti* tetrathyridia. (B) Proportions of novel and known genes harboring a signal peptide.
(TIF)

S1 Table Proteins exclusively detected in ES products from *M*. *corti* tetrathyridia but not in larvae somatic extracts. (XLSX)

S2 Table Proteins exclusively detected in somatic extracts from *M*. *corti* tetrathyridia but not in ES products. (XLSX)

S3 Table Proteins shared between the ES products and somatic extracts from *M*. *corti* tetrathyridia. (XLSX)

S4 Table Proteins overexpressed in the ES products from *M*. *corti* tetrathyridia. (XLSX)

S5 Table Proteins detected in fractions of *M*. *corti* tetrathyridia ES products (XLSX)

S6 Table Most represented proteins exclusively detected in active McES fractions (XLSX)
