# Supplementary figures and images for: Proteomic Analysis of Excretory-Secretory Products of Mesocestoides corti Metacestodes Reveals Potential Suppressors of Dendritic Cell Functions
Source: PLoS Negl Trop Dis. 2016 Oct 13;10(10):e0005061. doi: 10.1371/journal.pntd.0005061 (PMC5063416; doi:10.1371/journal.pntd.0005061)

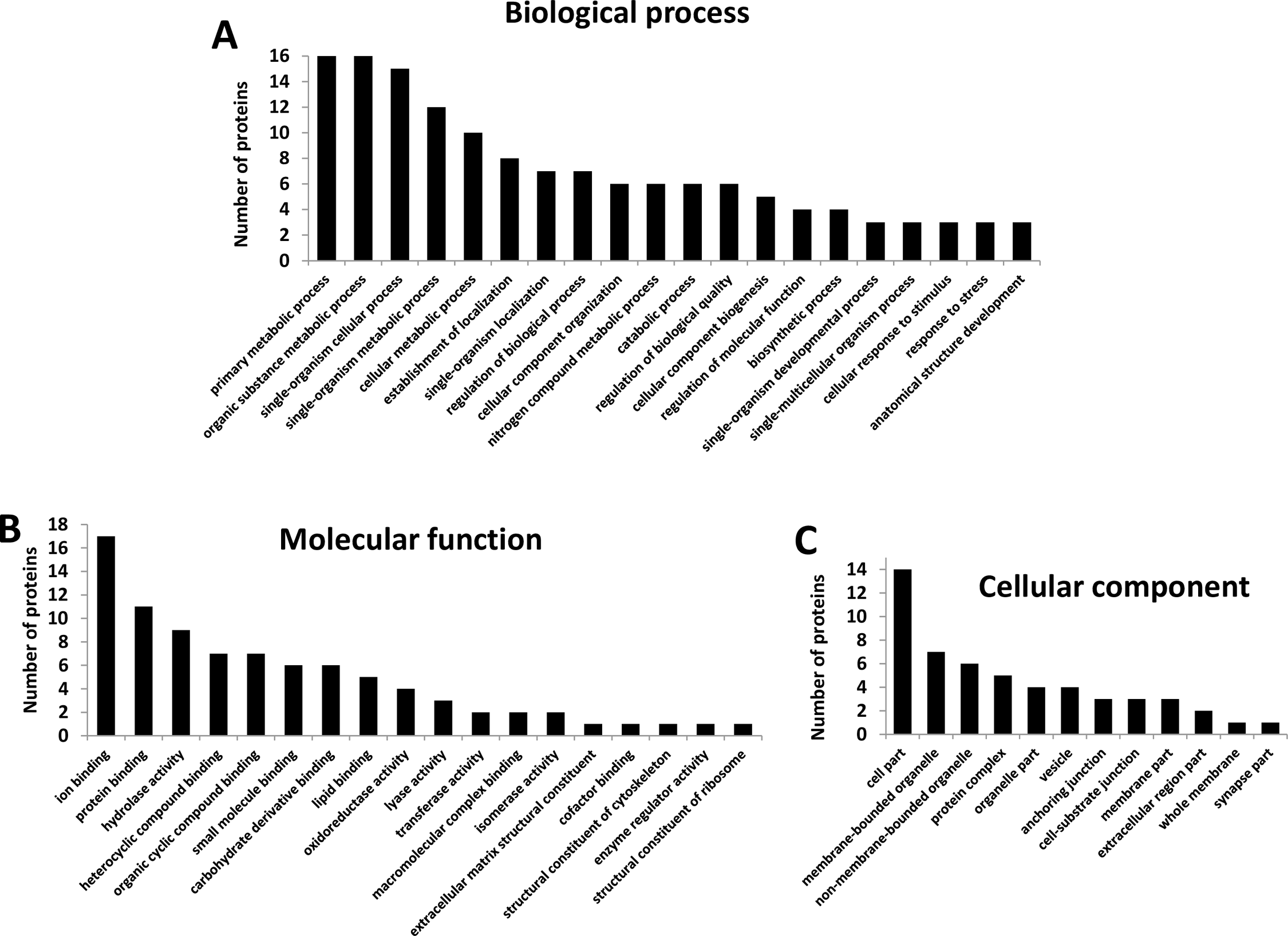

Supplement: S1 Fig — BLASTP searches were performed using Blast2GO against the NCBInr database. Most abundant GO terms for biological processes (A), molecular function (B) and cellular component (C) are shown. (TIF) [file pntd.0005061.s001.tif]

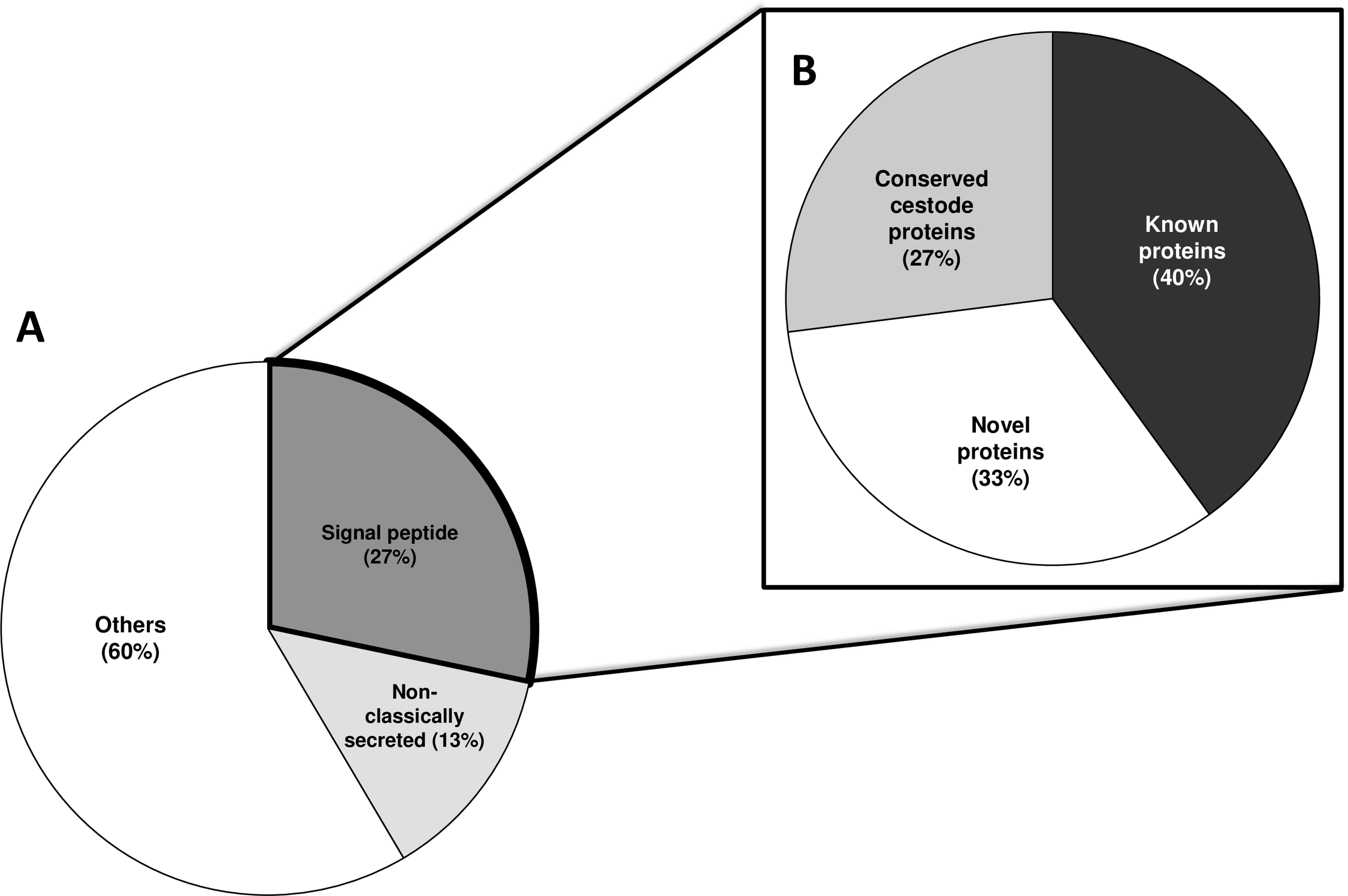

Supplement: S2 Fig — (A) Prediction of secretory pathways of proteins detected in ES products from M. corti tetrathyridia. (B) Proportions of novel and known genes harboring a signal peptide. (TIF) [file pntd.0005061.s002.tif]
